# Controlling the 3D Electromagnetic Coupling in Co-Sputtered Ag–SiO_2_ Nanomace Arrays by Lateral Sizes

**DOI:** 10.3390/nano8070493

**Published:** 2018-07-05

**Authors:** Fan Zhang, Shuang Guo, Yang Liu, Lei Chen, Yaxin Wang, Renxian Gao, Aonan Zhu, Xiaolong Zhang, Yongjun Zhang

**Affiliations:** 1 Key Laboratory of Functional Materials Physics and Chemistry of the Ministry of Education, Jilin Normal University, Changchun 130103, China; zhangfan147258@126.com (F.Z.); guoshuang0420@163.com (S.G.); ren7771993@163.com (R.G.); aonanzhu@163.com (A.Z.); zhangxiaolong10086@126.com (X.Z.); yjzhang@jlnu.edu.cn (Y.Z.); 2 College of Chemistry, Jilin Normal University, Siping 136000, China; 3 College of Physics, Jilin Normal University, Siping 136000, China

**Keywords:** co-sputtered, nanomace arrays, SERS, FDTD

## Abstract

Ag–SiO_2_ nanomace arrays were prepared on a two-dimensional ordered colloidal (2D) polystyrene sphere template by co-sputtering Ag and SiO_2_ in a magnetron sputtering system. The lateral size of the nanomaces and the distance between the neighbor nanomaces were controlled by adjusting the etching time of the 2D template. The nanomaces were composed of SiO_2_-isolated Ag nanoparticles, which produced surface-enhanced Raman scattering (SERS) enhancement, and 3D hot spots were created between the neighbor nanomaces. When the distance between the nanomaces was sufficiently large, triangle-shaped nanostructures on silicon substrate were observed, which also contributed to the enhancement of the SERS signals. The finite-difference time-domain (FDTD) method was used to calculate the electromagnetic field distributions in the Ag–SiO_2_ nanomace arrays, which generated physical reasons for the change of the SERS signals.

## 1. Introduction

Metal nanostructures have been studied for decades, mainly due to their unique properties and wide applications in some fields, for example, catalysis, electronics, optics, information storage, biological sensors, and surface-enhanced Raman scattering (SERS) [1,2,3,4]. In particular, people use Au, Ag, and other noble metals because their surface plasmon resonance frequency are in the visible range of the electromagnetic spectrum. When these particles are dispersed in a liquid medium, they can present a strong color, and the characteristics are attributed to the surface plasmon properties induced by the electromagnetic field [5,6]. The surface plasmon resonance of the nanoscale metal particle is different from that of the bulk material, and it has close relation with size, shape, agglomeration of particles, and the dielectric property of the surrounding media [7,8].

Since the SERS technique was developed in 1974, it has been used as a detection method of high sensitivity. SERS-active substrate preparation is the key factor, and the active, stable, reusable substrate is of great significance for application and basic research. Numerous SERS-active substrates have been developed, such as metal colloidal solutions, irregular metal films, and nanoparticles [9,10,11,12,13,14,15]. In addition, there are other non-traditional nanostructures, for example, nanotips, nanowires, nanorings, nanobowls, etc. [16,17,18,19,20,21,22,23]. Although chemical methods are commonly used to prepare noble metal nanocrystals, the size of the particles is limited, the surface is easily oxidized, and the biological toxicity limits its usages [24,25]. Silica-based nanocomposites have been widely studied due to some important properties. As an ideal support for nanomaterials, silica is generally thermally stable, water-soluble, nontoxic, biocompatible, and has high colloidal stability [26,27]. Composite metal nanostructures can be used as a SERS platform to provide great electromagnetic field enhancement, because the superior properties of composite nanostructures are allocated to single component [28,29]. Our group has reported ordered nanocap arrays composed of SiO_2_-isolated Ag islands on 2D polystyrene arrays. When the thickness of SiO_2_–Ag is 40 nm, the surface roughness is maximized, and the nanogap is narrowest, SERS enhancement is obtained [30]. 

Here, we co-sputtered silver and silica to prepare Ag–SiO_2_ nanomace arrays with a sizeable 3D hotspot distribution. When the surface roughness is constant, the maximum SERS enhancement is achieved by changing the nanogaps. When the nanogap size between the nanomaces is large enough, the triangle-shaped nanostructures between the nanomaces contribute to the enhancement of the SERS signals. Finite-difference time-domain (FDTD) simulation results show the local electric field distribution of the Ag–SiO_2_ nanomace arrays and provide the physical reasons for the obtained SERS observations. 

## 2. Experimental Section

### 2.1. Materials

Monodisperse polystyrene (PS, 200 nm) colloid particles were purchased from Duke Scientific Corporation, Palo Alto, CA 94303, USA (the density is 1.05 g/cm^3^). Silicon wafers, 4-mercaptobenzoic acid (MBA), sodium dodecyl sulfate, NH_3_·H_2_O, H_2_O_2_, and ethanol were purchased from Sigma-Aldrich Co., Ltd., Beijing, China. Ag and SiO_2_ targets were purchased from Beijing Jing Mai Mstar Technology Co., Ltd., Beijing, China. Deionized water (18.0 MΩ·cm^−1^) and ethanol were used throughout the entire experimental process.

### 2.2. Assemble of PS Sphere Arrays

We prepared PS sphere arrays by a self-assembly technique. The silicon substrates were boiled in H_2_O_2_, NH_4_OH, and H_2_O mixed solution (volume ratio 1:2:6) for 15 min, and the Si substrates were immersed alternatively in deionized water and alcohol ultrasonically for 10–15 min three times. Then, Si substrates were kept in deionized water. Alcohol and PS spheres were mixed with a volume ratio of 1:1. Subsequently, we dropped the mixture onto dry Si wafers, and then slowly immersed the wafers into the deionized water. Ordered monolayer films were formed on the water surface. The ordered monolayer is made up of films with Si substrates. Finally, the monolayer film of the 2D PS arrays was formed on the Si wafers.

### 2.3. Preparation of Ag–SiO_2_ Nanomace Arrays and Probe Molecule Absorption

In order to obtain Ag–SiO_2_ nanomace arrays with different sizes, PS bead arrays were etched for 0 s, 60 s, 90 s, and 120 s by a gas mixture of oxygen (25%) and argon (75%). Subsequently, PS bead arrays were used as templates for co-sputtering Ag and SiO_2_ in a magnetron sputtering system with a pressure of 0.6 Pa. Finally, we obtained the Ag–SiO_2_ nanomace arrays, and the film thickness was 130 nm. Then, these substrates were immersed in tetrahydrofuran solution to remove the PS colloidal particles. Double-sided adhesive tape was used to transfer nanomace arrays to another silicon wafer with the bottom up.

The concentration of 4-MBA probe molecules was 10^−3^ mol/L. The Ag–SiO_2_ nanomace arrays were immersed in 4-MBA for 30 min and then washed thoroughly with ethanol to remove unabsorbed probe molecules. 

### 2.4. Characterization of Substrates and SERS

Scanning electron microscope (SEM) images (JEOL-6500F, JEOL LTD., Tokyo, Japan) were produced using an acceleration voltage of 5.0 kV. Transmission electron microscope (TEM) images (JEM-2100F, JEOL LTD., Tokyo, Japan) were obtained using an acceleration voltage of 200 kV. Raman spectra were measured by a Renishaw Raman system, model 2000 confocal microscopy spectrometer (model 2000, Renishaw, London, UK) with an excitation wavelength of 514.5 nm (40 mW, power out of 1%). The microscope was used to focus the laser beam onto a spot of diameter 1 μm with a 50× long-range objective. The equipment was in a 180° backscattering geometry and the time to collecting the signal was set at 10 s. X-ray photoelectron spectroscopy (XPS) was conducted with a ESCALAB-MARKII spectrometer (Thermo Fisher Scientific, Waltham, MA, USA) employing an Al-Kα X-ray source. 

### 2.5. Finite-Difference Time-Domain (FDTD) Simulations 

Commercial FDTD (Lumerical FDTD solution) was used to simulate the electromagnetic field distribution in Ag–SiO_2_ nanomace arrays. The incident light wavelength was 514.5 nm. The polarization direction was parallel to the X axis. The geometric parameters of modes came from the top and cross-sectional views of SEM images, periodic boundary conditions were 200 nm, and mesh size was 1 nm; a frequency monitor was used to collect the electromagnetic field distribution of Ag–SiO_2_ nanomace arrays. The refractive index of the Ag–SiO_2_ complex films were measured by ellipsometer, and the reflected index of PS sphere was 1.585 [31].

## 3. Results and Discussion

**Figure 1:**
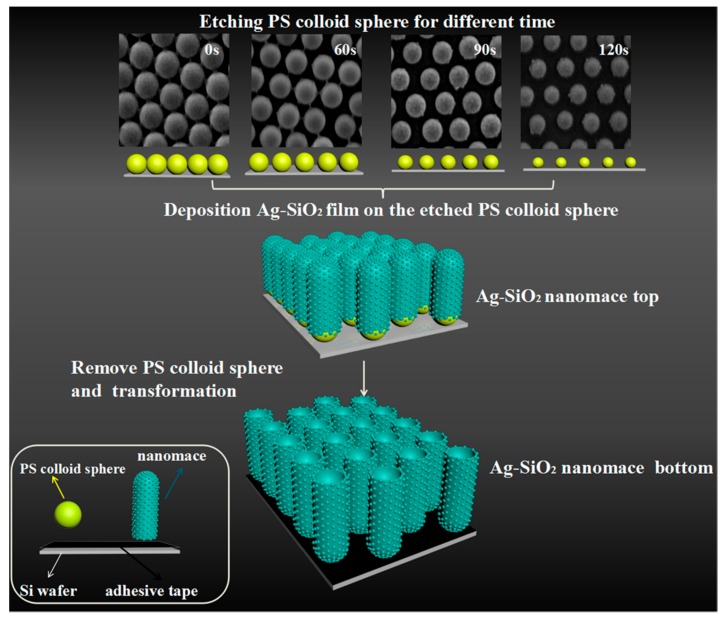
Schematic diagram of the fabrication procedure for Ag–SiO_2_ nanomace arrays.

The preparation process of Ag–SiO_2_ nanomace arrays is illustrated in Figure 1. Firstly, the two-dimensional (2D) ordered colloid polystyrene (PS) bead arrays are prepared by a self-assembly technique. Then, the colloidal beads are etched for different times to obtain the controlled arrays with different diameters and distances between the neighboring PS beads. Figure 1 shows the SEM images of 2D PS templates after etching for different times. The PS beads without etching were closely packed with smooth surfaces, and the average diameter was 200 nm. With increasing etching time, the distance between beads becomes larger and the size of beads decreases, but the period and morphology of arrays remain unchanged, which indicates PS bead arrays on the substrates are stable. The average diameters change from 165 nm for etching for 60 s to 130 nm for etching for 120 s. The PS beads maintain the spherical shape, although the surface roughness slightly increases when the etching time is over 90 s. After that, the Ag and SiO_2_ targets are co-sputtered onto the colloidal substrates to form the nanomace arrays. The nanomace arrays are transferred to another Si substrate with double-sided adhesive tape, and the PS colloidal beads are etched by tetrahydrofuran solution for 15 min, forming the Ag–SiO_2_ bottoms.

**Figure 2:**
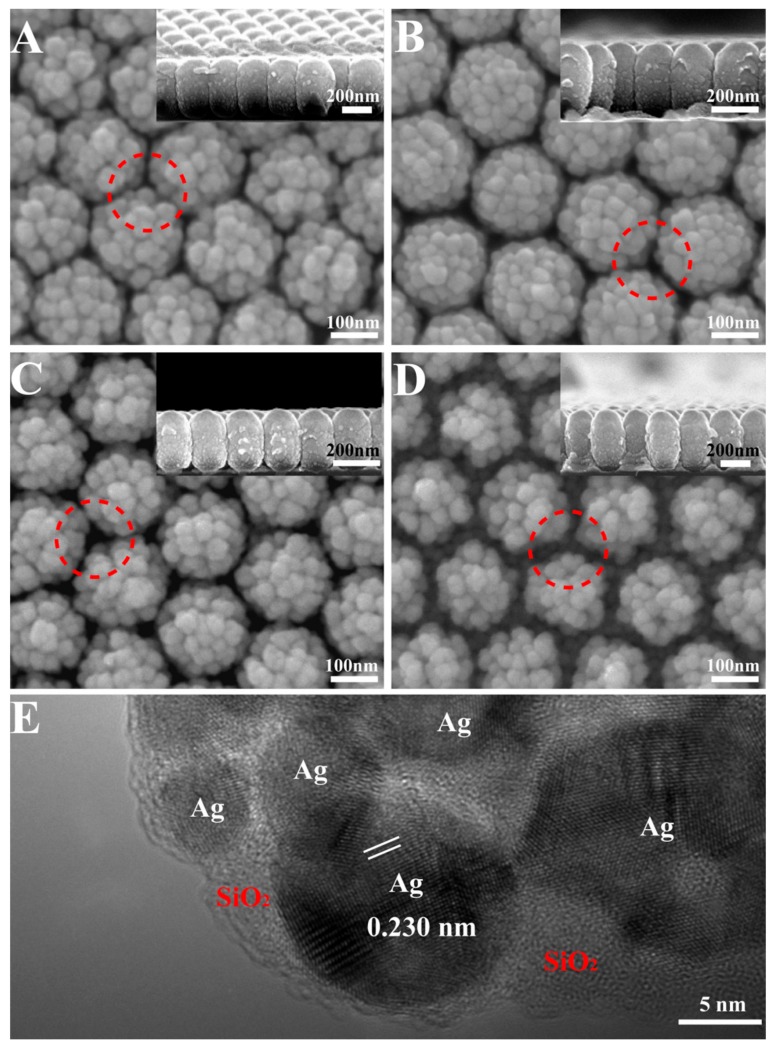
Top-view SEM (**A**–**D**) images of the Ag–SiO_2_ nanomace arrays with etching for (**A**) 0 s; (**B**) 60 s; (**C**) 90 s; (**D**) 120 s. HRTEM image (**E**) of the Ag–SiO_2_ nanomace arrays. The illustrations are of cross-sections of the samples.

Figure 2 shows the SEM and High resolution transmission electron microscope (HRTEM) images of the co-sputtered Ag–SiO_2_ films with different etching times (A–D). The films are composed of compact nanometer units with curved surfaces. Although the curved units show different lateral sizes, the Ag–SiO_2_ particles show a uniform size among the different samples in Figure 2, which indicates the morphology of different samples is determined by the composition of the films, and the PS bead sizes have little effect on the surface morphology of the units. The lateral size of the units is 200 nm for the samples without etching (Figure 2A), 190 nm after etching for 60 s (Figure 2B), 185 nm for 90 s (Figure 2C), and 170 nm for 120 s (Figure 2D). The results above show that an increase in the distances between the neighboring units reduces the restriction to lateral growth. Without etching, the nanomaces are connected to each other. When etching is performed on the PS substrates, the obtained nanomaces are separated from their neighbors. The nanogaps gradually increase, as shown by the red dotted circles. For the samples based on PS etched for 60 s and 90 s in Figure 2D,C, the average distance between the neighboring nanomaces is 10 nm and 15 nm. When the PS is etched for 120 s, the average distance between the neighboring nanomaces is 30 nm, and Ag–SiO_2_ deposition is clearly observed on the substrates, even in the top-view image. The section-view images in the inset show that the units are composed of protruding particles around rod-like shapes, and we named these nanoscale units nanomaces. The section-view images also show the changes in substrates for different deposition times. For the samples on the PS arrays without etching in Figure 2A, no obvious materials are observed on substrates because of the shadow effect. The lateral size of each unit is about 200 nm, in agreement with the PS size, while the vertical size is around 330 nm, which indicates anisotropic growth due to the shadow effect by the neighboring units and the tilt deposition. For the samples etched for 60–90 s, the samples on the PS beads show obvious anisotropic growth, and triangular islands are observed on the Si substrates between three neighboring PS beads, which indicates that the shadow effects are reduced, because the distance between the neighboring PS beads becomes larger after the etching process. As the etching time increases (Figure 2D illustration), the triangular islands grow gradually, but their heights are far smaller than the heights of the nanomaces. Every nanomace is composed of small particles, confirmed by HRTEM to be Ag–SiO_2_ particles. In Figure 2E, the HRTEM image shows that the distance of the lattice fringes is approximately 0.230 nm, which is in agreement with the (111) plane of Ag. The surrounding semitransparent areas are amorphous SiO_2_ [32], which was confirmed by XPS (Figure S1). 

**Figure 3:**
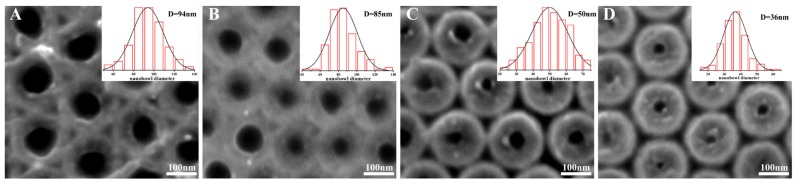
Top-view SEM images of nanomace arrays when transferred to a new Si substrate. The nanoholes with different sizes are observed when the PS templates are etched for (**A**) 0 s; (**B**) 60 s; (**C**) 90 s; (**D**) 120 s.

The bottom images of the Ag–SiO_2_ nanomace arrays were characterized by SEM, as demonstrated in Figure 3A–D. The images show the bottoms of the samples are smoother than the tops; this is because the film is very thin around the nanomaces bottom and the particles are very small. When the PS beads are etched for less than 60 s (Figure 3A,B), the bottoms of the nanomaces are connected. Triangle-shaped nanoholes are observed for the samples based on PS arrays without etching in Figure 3A, because the shadow effect prevents nanostructure formation on the substrates through the triangle-shaped nanoholes between three neighboring PS beads. When the PS beads are etched for more than 90 s (Figure 3C,D), the bottoms of the nanomaces are isolated completely. For all the samples, the bottoms of the nanomaces touching the substrates are left as nanoholes in the film. The sizes of the nanoholes are 94 nm, 85 nm, 50 nm, and 36 nm for the films based on the PS sphere arrays after etching for 0 s, 60 s, 90 s, and 120 s, respectively.

**Figure 4:**
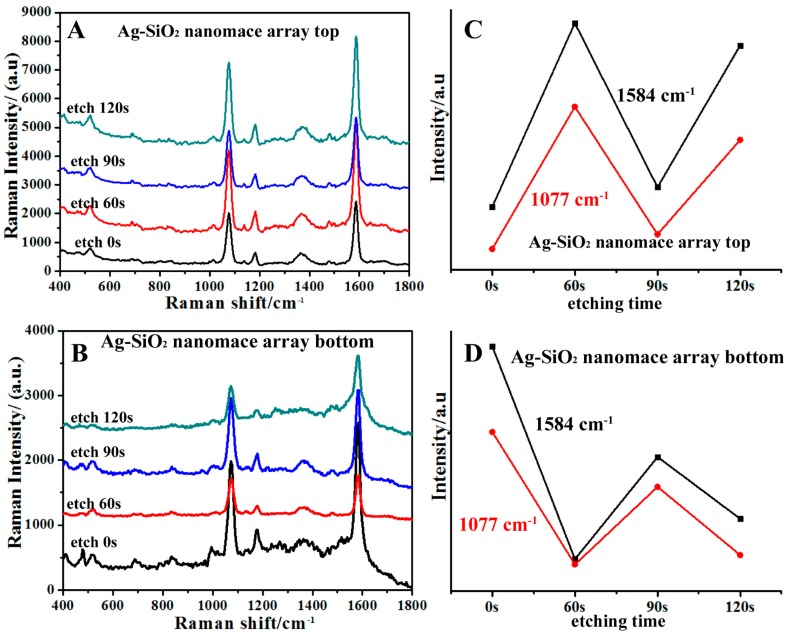
Raman spectra of top (**A**) and bottom (**B**) of nanomace array substrates; (**C**) and (**D**) show the corresponding dependence of the Raman signal intensity on etching times.

Figure 4 shows that the characteristic peaks of 4-mercaptobenzoic acid (MBA) probe molecules are observed for all samples. The peaks at 1077 cm^−1^, 1182 cm^−1^ and 1133 cm^−1^, 1362 cm^−1^, 1481 cm^−1^, and 1584 cm^−1^ are attributed to υ(C–S) ring-breathing mode, δ(CH) mode, υ(CC)(COO–) mode, υ(CC)+α(CH) combination mode, and aromatic υ(CC) mode of Raman spectra, respectively [33,34,35]. For the top of the nanomaces on the substrates without etching in Figure 4A, obvious SERS signals are observed because of the significant roughness caused by the SiO_2_-isolated Ag nanoparticles, in which the local surface plasmon resonances (LSPR) contributes to SERS enhancement. When etching time is performed for 60 s, nanogaps of around 10 nm are created between neighboring nanomaces, which also contributes to the SERS observations. Therefore, the SERS signals are enhanced more when etching occurs for 60 s. When PS beads are etched for 90 s, a large gap around 15 nm is created between the nanomaces, which reduces the electromagnetic coupling between the neighboring nanomaces and decreases the SERS observations. When PS beads are etched for 120 s, a larger gap is created between the nanomaces, which decreases the electromagnetic coupling intensity between the neighboring nanomaces. However, when the triangular nanostructures are exposed to SERS measurement and the triangles are large enough to couple with neighboring nanomaces, the SERS measurement is increased again. SERS measurements were also performed for the bottom of the nanomace arrays to determine their LSPR properties. For the bottoms of the nanomaces in Figure 4B,D, the samples without etching show an increased SERS signal compared to the samples on PS after etching for 60 s, because the contribution from the triangle-shaped nanoholes are screened out. The SERS observations of the samples on PS after etching for 90 s are enhanced a little, because the contributions come from the nanogap formation between the nanomaces, in addition to the nanogaps in the nanomaces. Decreased SERS signal is observed for the samples on PS after etching for 120 s because the distance of the nanogaps between the nanomaces increases, which leads to the reduced the coupling between the neighboring nanomaces. In summary, the SERS signal of the nanomace array bottoms is lower than the nanomace array tops. This is because the top of the nanomace arrays are rougher than the bottom, and the coupling between triangle-shaped nanostructures and nanomace arrays disappears. The nanogaps and roughness are important factors affecting SERS enhancement.

Further quantification of the SERS enhancement of Ag–SiO_2_ nanomace composite arrays was performed. The SERS enhancement factor (EF) [33,34] is calculated through the following equation: $EF=\left(I_{SERS}\times N_{bulk}\right)/\left(I_{bulk}\times N_{SERS}\right)$, while *I_SERS_* and *I_bluk_* are the SERS intensity of nanomace arrays and the solid 4-MBA (Figure S2) at 1584 cm^−1^. $N_{SERS}=N_{d}A_{laser}A_{N}/\delta$ is the number of 4-MBA molecules on the Ag–SiO_2_ nanomace composite arrays; δ is the area of a 4-MBA probe molecule about 0.33 nm^2^ [36]. N_d_ is the density of a PS sphere with a diameter of 200 nm, and *A_laser_* is the area of the focal laser spot. *A_N_* is the half surface area of a PS colloid sphere. $N_{bulk}=\rho A_{laser}hN_{A}/M$ is the number of solid 4-MBA molecules. The laser spot diameter is 1 μm. The density of 4-MBA (ρ) is 1.50 g/cm^3^, the molecular weight (*M*) is 154.19 g/mol, and the effective focused depth (*h*) is 19 μm [36]. So, the values of *N_bulk_* and *N_SERS_* are 8.73 × 10^10^ and 4.76 × 10^6^. The values of *I_SERS_/I_bulk_* are 4.9, 8.8, 5.3, and 8.12 for nanomace arrays with PS spheres etched for 0 s, 60 s, 90 s, and 120 s. The values of *EF* were calculated to be 8.97 × 10^4^, 1.61 × 10^5^, 9.67 × 10^4^, and 1.49 × 10^5^. The surfaces of nanomaces contain nanoparticle-rough surfaces, which increases the surface area. The surface area contributes to SERS enhancement, but it is not the main factor [30].

**Figure 5:**
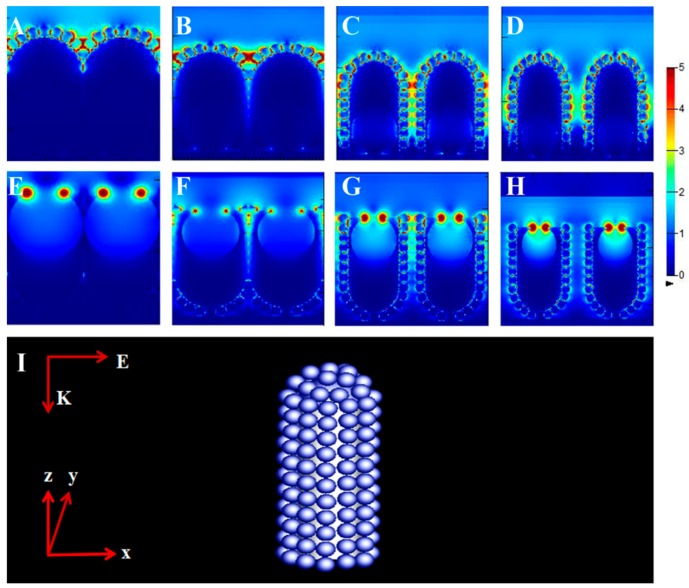
The finite-difference time-domain (FDTD) simulation of the tops and bottoms of nanomace arrays, with PS spheres etched for (**A**,**E**) 0 s; (**B**,**F**) 60 s; (**C**,**G**) 90 s; (**D**,**H**) 120 s. (**I**) The FDTD model of Ag–SiO_2_ nanomace arrays.

In order to analyze the distribution of the electromagnetic (EM) field of the nanomace arrays, an FDTD simulation was used to calculate the local field enhancement of the nanomace arrays in Figure 5. In Figure 5A, the “hot spots” are located in the nanogaps between the nanoparticles on the nanomace tops. In Figure 5B, the simulation reveals that the additional contribution from the coupling between the nanomaces enhances the SERS signal. When the sizes of PS spheres become smaller, every nanomace diameter decreases, and the nanogaps between nanomaces reach optimal distances. When the nanogaps increase further, the distance between nanomaces becomes larger and the coupling becomes smaller. The simulation in Figure 5C shows that the coupling fields are reduced between the nanoparticles on the nanomace tops. However, the heights and widths of the triangular islands in Figure 5D are so large that obvious electromagnetic coupling occurs between the triangular islands and the neighboring nanomaces, which tends to enhance the local electromagnetic field. In order to analyze the contribution of the bottom of the PS spheres to the EM field of the nanomaces, we simulated the bottoms of nanomaces; the models are shown in Figure 5E–H. All the figures show that the “hot spots” are localized around the nanohole mouth and there are no significant effects on the SERS contributions from the nanomaces. When the light is incident from the nanomace tops, the contributions from the nanohole mouth are reduced greatly. The growth of the triangular structure between the nanomaces changes the collective electron movement, and the strong coupling is transferred from the nanohole mouth, which is also responsible for the increased SERS signal observed in Figure 5D. Figure 5I shows the simulation mode of Ag–SiO_2_ nanomace arrays without etching.

## 4. Conclusions

In summary, we prepared Ag–SiO_2_ nanomace arrays by magnetron sputtering on 2D PS sphere arrays. This process focuses on regular and controlled “hot spots” and compares EM field distribution between nanocaps and nanoholes. A lot of “hot spots” are created by controlling the lateral size of the nanomaces. A 4-MBA solution was used as probe molecules to investigate the SERS property. The results show that the SERS signal from each nanomace is nearly the same due to uniform roughness. The electromagnetic coupling between the nanomaces can be tuned by controlling the distance between neighboring units. The nanohole at the nanomace bottom and the triangle-shaped nanostructures on the silicon substrates also contribute to SERS enhancement when the distances are large enough. The preparation method is simple, less time-consuming, and low cost, with good controllability, a high repetition rate, and perfect stability.

## Supplementary Materials

The following are available online at http://www.mdpi.com/2079-4991/8/7/493/s1, Figure S1: XPS spectra of (A) the full-scan spectrum; (B) Si 2p; (C) Ag 3d of SiO_2_-Ag nanomace arrays, Figure S2: Raman spectra of solid 4-MBA excitation with wavelength was 514.5 nm (40 mW, power out of 1%).
