# Supplementary material for: Controlling the 3D Electromagnetic Coupling in Co-Sputtered Ag–SiO2 Nanomace Arrays by Lateral Sizes
Source: Nanomaterials (Basel). 2018 Jul 5;8(7):493. doi: 10.3390/nano8070493 (PMC6071140; doi:10.3390/nano8070493)
Supplement: Supplementary file 1 [file nanomaterials-08-00493-s001.pdf]

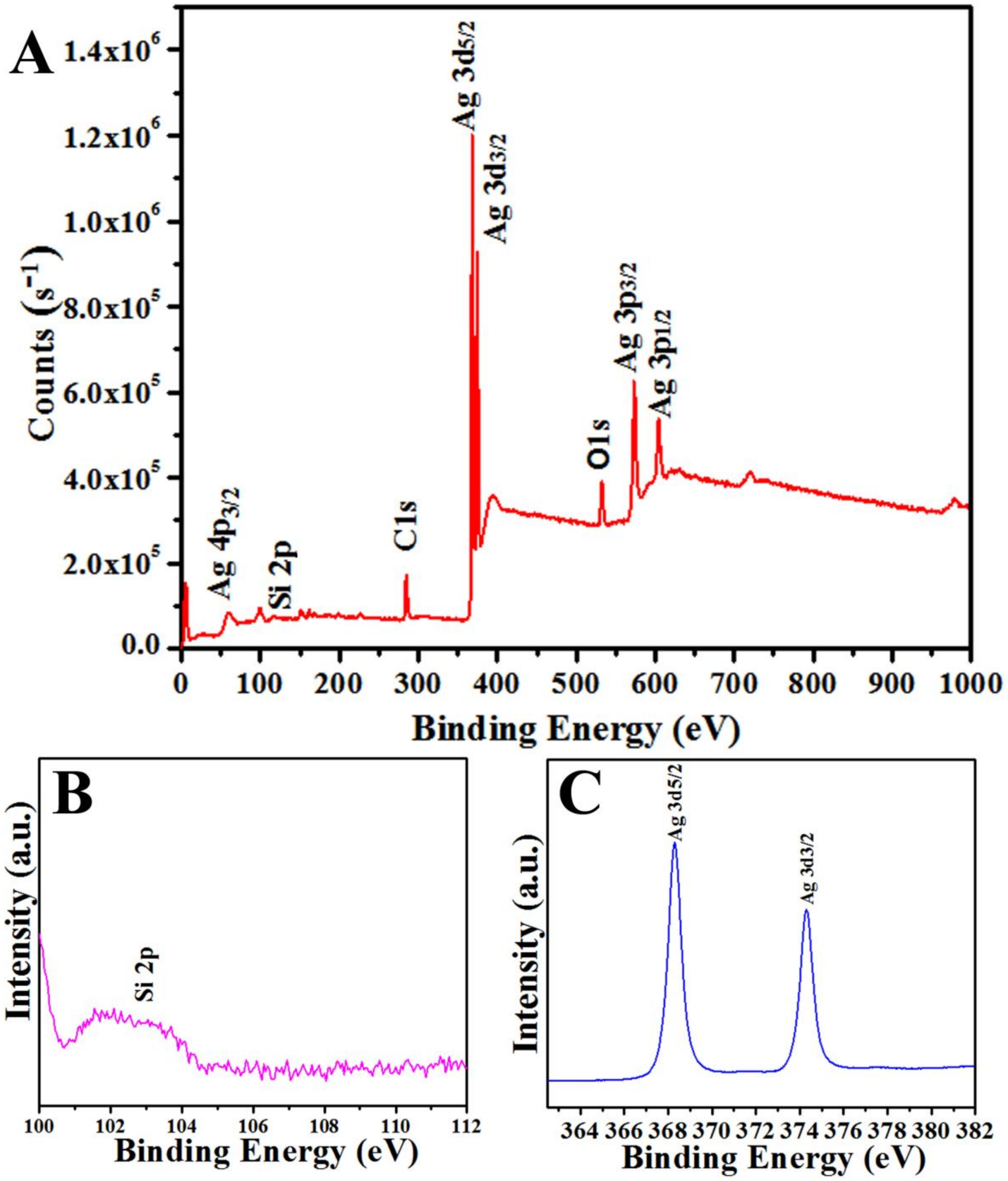

Figure S1. XPS spectra of (**A**) the full-scan spectrum; (**B**) Si 2p; (**C**)Ag 3d of SiO_2-Ag nanomace arrays.

X-ray photoelectron spectroscopy (XPS) is used to analyze the compositions and chemical bonding states of Ag-SiO_2 nanomace arrays in Figure S1. Figure S1A shows the peaks of Ag, Si, C and O elements in the full-scan spectrum. As shown in Figure S1B, the peak of Si 2p at about 103.0 eV can be observed, indicating the formation of the Si-O-Si bond [1]. And the speak of O 1s located at 532.6 eV in Figure S1A, which is consistent with the binding energies for O 1s peaks obtained from SiO_2 [2]. Figure S1C shows the two binding energy of Ag 3d $_{5/2}$ and Ag 3d $_{3/2}$, which can be assigned to peak of 368.27 and 374.28 eV, respectively.

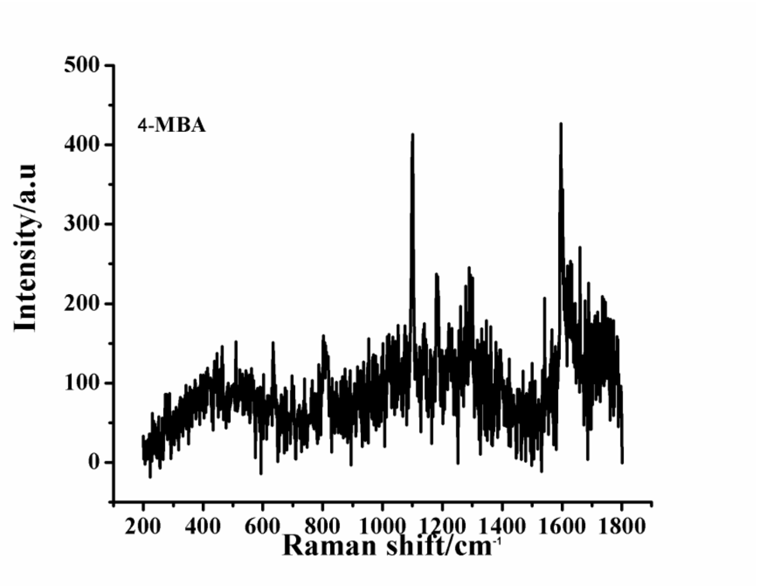

Figure S2. Raman spectra of solid 4-MBA excitation with wavelength was 514.5 nm (40 mW, power out of 1%).
